# Does the EU COVID Digital Certificate Strike a Reasonable Balance between Mobility Needs and Public Health?

**DOI:** 10.3390/medicina57101077

**Published:** 2021-10-09

**Authors:** Gianluca Montanari Vergallo, Simona Zaami, Francesca Negro, Pietro Brunetti, Alessandro Del Rio, Enrico Marinelli

**Affiliations:** 1 Department of Anatomical, Histological, Forensic and Orthopedic Sciences, Sapienza University of Rome, 00161 Rome, Italy; gianluca.montanarivergallo@uniroma1.it (G.M.V.); francesca.negro@uniroma1.it (F.N.); alessandro.delrio@uniroma1.it (A.D.R.); enrico.marinelli@uniroma1.it (E.M.); 2 Unit of Forensic Toxicology, Section of Legal Medicine, Department of Excellence of Biomedical Sciences and Public Health, Marche Polytechnic University of Ancona, 60126 Ancona, Italy; pietrobrunetti40@gmail.com

**Keywords:** COVID-19, vaccine, COVID digital certificate, EU regulation, proportionality, privacy, freedom of movement, public health

## Abstract

The need to fight a highly aggressive virus such as SARS-CoV-2 has compelled governments to put in place measures, which, in the name of health protection, have constrained many freedoms we all enjoy, including freedom of movement, both nationally and within the European Union. In order to encourage and facilitate the return to free movement, the European Parliament has launched a “COVID-19 digital certificate”. A spirited debate centered around the use of this certificate is still ongoing among scholars, many of whom have pointed out the uncertainties relative to COVID-19 immunity, privacy issues and the risk of discriminatory effects. The authors, while highlighting some critical aspects, argue that the COVID digital certificate in its current approved version can effectively help prevent the spread of the infection and promote free movement, while upholding the right to health as much as possible. However, they also stress the need for a thorough information campaign to illustrate the advantages and limitations of this document in order to avoid creating a false sense of security in the public opinion, who may wrongly assume that the emergency has been overcome for good.

## 1. Introduction: The Role of the European Union in Terms of Managing Free Movement during the Pandemic

The COVID-19 pandemic has triggered a global health emergency, by infecting 63,091,712 people, with 1,248,038 deaths in Europe alone, as of 18 August 2021 [1]. Moreover, the infection has led to strained, overcrowded hospitals, which has caused dangerous delays in the care and treatment of other diseases [2,3]. In the first phase of the emergency, the European Union has appeared more concerned with protecting public health than with upholding freedom of movement or the stability of the Schengen system [4]. Consequently, the EU has allowed Member States to introduce measures aimed at restricting free movement within the Union, by requiring, for instance, those returning from certain countries, including EU countries, to produce a certificate documenting a negative test result or by imposing quarantine. On 12 August 2020, for example, the Italian government via the Ministry of Health issued a decree requiring travelers from Croatia, Greece, Malta and Spain to produce a negative test result on their return into the country, while remaining under home isolation, although it is rather unclear how to comply with such requirements and who should provide oversight. Other EU nations have chosen to ban citizens of certain countries from entering their territory: this is the case of Bulgaria, for example, whose government on 28 July 2020 phased in restrictions to travelers from Italy, Spain, France, Germany and some other countries heavily affected by the pandemic [5]. The restrictive measures have produced a decrease in infections, which has led the European institutions to revoke or loosen the measures limiting freedom of movement [6]. Unfortunately, despite the travel restrictions at the national level, the 2020 summer spent in conditions of near normalcy has caused the infection rate to rise again. Hence, in the autumn of 2020, a “second wave” of the disease broke out, exacerbated by the spread of the variants. In fact, “during December 2020, an unexpected increase in the number of COVID-19 cases in the United Kingdom and South Africa was found to be associated with the emergence of the new SARS-CoV-2 variants of concern (VOC) 501Y.V1 (B.1.1.7) and 501Y.V2 (B.1.351), respectively. Such variants had mutations (N501Y) at the receptor-binding domain of the spike protein associated with an apparent increase in viral transmission of 40–70%, and possibly an even higher degree of clinical severity [7]”. At the beginning of 2021, such variants spread further, forcing the implementation of new air travel restrictions [8].

Outside of the European Union, Israel was the first country to move in the direction of establishing a “green pass”, introduced on 21 February 2021 for vaccinated or fully recovered individuals as part of a set of incentive/penalization measures related to vaccination, such as the exemption from quarantine and access to cultural, social and sporting events [9]. Against such a backdrop, some southern European member states, such as Cyprus, Greece, Portugal, Spain and Italy, got worried about the risk of harming the summer hospitality industry, which is essential for their national economies, and have themselves devised COVID passes that can grant bearers exemption from restrictions [10]. These passes enabled the tourism industry to restart along with traveling, in particular airline traffic, which had suffered the most due to the obvious difficulty in guaranteeing compliance with distancing requirements in small and closed spaces. Such a certificate was valid for travel on the national territory. Therefore, in order to travel abroad, people still had to adhere to the national regulations of each individual country of destination, which still required, depending on the case, a negative test carried out in the hours prior to departure, or quarantine upon arrival [11,12].

It is fairly obvious that if each state chooses the type of document certifying the vaccination status, a negative test or recovery, it becomes all but impossible to achieve a European standardization, which should instead be a primary objective in consideration of the pandemic nature of the COVID-19 emergency [13]. Furthermore, the various different national passes have created difficulties in ascertaining their validity when traveling within the EU. It is no coincidence that Interpol has reported cases of illicit sales of counterfeit negative COVID-19 test certificates, both online and offline, involving organized forgery groups [14].

## 2. COVID Digital Certificate’s Distinctive Traits

Aiming for a European common strategy for managing the health crisis, on 14 June 2021, the European Parliament approved the “EU Digital COVID Certificate” [15], the result of lengthy negotiations among Member States. The Regulation is binding and directly applicable throughout the European Union from 1 July to 30 June 2022. It is not a “passport” in the strict sense (Article 3 paragraph 5), because the principle of free movement must be always guaranteed, but rather a tool designed to facilitate movements within the EU during the COVID-19 pandemic and prevent individual states from putting in place uneven and discriminatory measures (Article 1). The COVID Digital Certificate allows EU citizens and residents to move more easily, avoiding the restrictions that individual states could independently impose, such as quarantine or mandatory testing. According to the newly approved rules, EU states will not be able to impose further travel restrictions on certificate holders—such as quarantine, self-isolation or testing—“unless they are necessary and proportionate to safeguard public health” and, in that case, they will have to keep account of the scientific evidence, “including epidemiological data published by the European Center for the Prevention and Control of Diseases” (art.11 co.1). The state that decides to adopt the aforementioned restrictions must inform the Commission and the other member states 48 h prior to the entry into force of such new rules (Article 11 paragraph 2). The underlying assumption is that the free movement of those deemed immune to SARS-CoV-2 and who cannot transmit the disease should not be restricted, since such a penalization is not necessary to achieve the ultimate goal: the protection of public health in compliance with the principles of proportionality, subsidiarity and non-discrimination.

## 3. Main Characteristics of COVID Passes

The document includes three types of certificates: vaccination certificates, test certificates and recovery certificates (Article 3). Specifically, the vaccination certificate, which documents the successful administration of an anti-COVID-19 vaccine, must contain the name of the holder of the vaccine patent, the number of doses administered and the date of the last dose received. (Article 5, Annex 1). Individual states may possibly consider valid the certificate showing vaccination with other vaccines not approved by the European Drug Authority (e.g., the Russian Sputnik). In this case, they must accept the relevant certificates issued in other Member States. The test certificate has to document the result of a PCR test or a rapid antigen test among those included in a special list agreed on within the Health Security Committee on 17 February 2021 [16]. Such a document must report the type and name of the test, the date, the time, the facility in which the test was carried out and the result found (Article 1 paragraph 3). Finally, the certificate of recovery proves that the holder of the certificate has recovered from a SARS-CoV-2 infection. This certificate can be issued no earlier than eleven days after the date on which the person underwent a test certifying recovery (art. 7) and is valid up to a maximum of 180 days after the test. Both tests must be carried out by healthcare professionals or qualified personnel in the Member State issuing the certificate. The COVID Digital Certificate must be issued to all European citizens, including citizens of cross-border states, and therefore, also to citizens or residents of the Principality of Monaco, the Vatican City, the Republic of San Marino and the Principality of Andorra, regardless of the state in which they were vaccinated, as long as it is an European Medicines Agency (EMA)-approved vaccine. For other countries, it will be up to the Commission to establish whether the certificates of an extra-EU country comply with the guarantees of the Union regulatory framework and whether that country in turn acknowledges the certificates issued by the Union under conditions of reciprocity [17]. The regulation also requires that the COVID Digital Certificate must be issued free of charge, in digital or paper format, at the choice of the holder, or in both formats to facilitate “persons with limited access to digital technologies.” It must have a QR code that certifies its authenticity. The COVID Digital Certificate is interoperable at the European level through the technological systems internationally established (Article 4). In order to facilitate European interoperability, the certificate must be drawn up at least in the official language (or languages) of the issuing member state and in English, reporting the information in a clear and legible format (Article 3 paragraph 2). The Commission will set up a gateway to ensure that certificates can be verified throughout the EU and will help Member States in the technical implementation of certificates. The main essential characteristics of COVID passes are summarized in Table 1.

## 4. COVID Digital Certificate and Privacy Safeguards

There is no denying that the wide circulation of personal and health data limits the right to the protection of personal data, enshrined in art. 8 of the EU Charter of Fundamental Rights and, in more detail, by Regulation (EU) 2016/679. Of course, balancing the two rights, health and privacy, is not a simple operation. It is no coincidence that various documents approved within the Council of Europe and the European Union have established some fundamental points by which to strike such a balance in a manner consistent with the needs of a democratic society inspired by respect for the dignity of the person and human rights [18,19,20,21]. The principle that the regulation is meant to affirm is that the limitation of privacy is legitimate if it serves to safeguard a greater interest of public health and social security [22]. Already in the first months of the pandemic, tools for tracing contacts with positive subjects had been used to contain the spread of the disease, and since then, the thorny issue of balancing the right to health with the right to privacy had already manifested itself [23,24]. The regulation asserts that no personal data of the certificate holders will pass through the gateway, nor will it be stored by the Member State carrying out the verification (recital 16). In order to protect the privacy of the citizenry, “the unique certificate identifier is composed of an alphanumeric string and Member States should ensure that it does not contain any data linking it to other documents or identifiers, such as to passport or identity card numbers, in order to prevent directly identifying the holder. The unique certificate identifier should be used only for its intended purposes, which include requests for the issuance of a new certificate if a certificate is no longer available to the holder and the revocation of certificates. In addition, the use of a unique certificate identifier avoids the need to process other personal data that would otherwise be necessary to identify individual certificates” (Recital 19). Indeed, in adherence to the principle of minimization of processing provided for by EU Regulation 2016/679°, the COVID digital certificate must contain only the information for the issuance of the certificate itself, such as name, date, country of birth, date of issuance, a limited number of health data, relevant vaccine/test/recovery information and unique identifier. All health data are stored in the issuing Member State and may not be stored or retained when a certificate is verified in another Member State. For verification purposes, only the validity and authenticity of the certificate must be checked, ascertaining by whom it was issued and signed. The information contained in the certificate must be kept only for the time strictly necessary to serve its purpose, and by no means beyond the period during which the certificates can be used to exercise the right of free movement within the European Union (Article 10 co. 3 and 4). The measures laid out in the COVID Digital Certificate regulatory framework will be discontinued once the COVID-19 pandemic is over, since there will be no sensible reason to require citizens to submit health documents to exercise their right of free movement. By the same token, however, the regulation holds that the regulations will be put back in place if the WHO declares another pandemic due to the spread of COVID-19 or a variant thereof, or similar infectious diseases with a pandemic potential. Therefore, the effectiveness of the strategies to combat the epidemic are legitimate in principle, as they protect a relevant public interest, provided that they abide by the principles of proportionality and provisionality, i.e., they are applied in an emergency situation and only for the duration of the emergency [25].

## 5. COVID Digital Certificate between Freedom of Movement and Health Protection

By proposing three types of certification, the European legislator has introduced the possibility for EU citizens to document compliance with public health standards with methods other than vaccination. The COVID Digital Certificate guarantees equal freedom of movement not only to those who have not yet been able to get vaccinated or to those who, in any case, for health reasons, will not be able to get vaccinated, but also to those who have chosen not to. In fact, a citizen can in fact refuse to be vaccinated and/or tested: in such cases, they are not stripped of their freedom of movement, but must be subjected to restrictions such as quarantine or self-isolation. The COVID Digital Certificate does not, therefore, introduce any mandatory vaccination, in accordance with the indications of the Council of Europe Commission for Social Affairs, Health and Sustainable Development, which has remarked that the obligation is a useless, if not counterproductive, imposition, and instead calls on Member States to develop strategies based on trust and thorough communication, which is considered more effective than coercive methods [26,27].

## 6. Discussion

The historical context in which the COVID Digital Certificate is placed, characterized by the pandemic and globalization, sets it apart as a very significant historical innovation. Not surprisingly, this certificate has sparked mixed reactions [28], in the scientific community as well as among the general public. Some have argued in its favor, because it allows people not infected with SARS-CoV-2 to move without other restrictions [29], although such a position ought to be evaluated with a grain of salt, given the fact that vaccines undoubtedly provide invaluable protection against severe COVID-19, but vaccinated individuals may still contract the infection and pass it on to others, as in cases of so-called vaccine breakthrough infections [30]. Currently, there are not enough studies to quantify such a risk, particularly in regards to the Delta variant. Those opposed to the certification system, on the other hand, point to the allegedly higher risk of discrimination that such a system may bring about. In fact, global repercussions are feared for middle and low-income countries, unable to carry out mass vaccination and consequently, inevitably discriminated against [31,32]. The COVID Digital Certificate could lead to discrimination when, for example, the vaccine doses available are not enough to treat the entire population. In this case, there would be a potential discrimination linked to the inequality between those who have had the opportunity to get vaccinated and those who have not. Lingering doubts such as the length of vaccine-induced immunity and, consequently, the appropriate validity of COVID passes issued after vaccination are still far from being clarified. According to EU sources, there is no maximum validity as yet determined for vaccination certificates, as that time span will depend on emerging scientific evidence pertaining to the length of protection of each vaccine available [33]. As a result, Belgium [34] and Switzerland [35] (whose COVID certificate is recognized by the EU/EFTA states and can, therefore, be used throughout the EU and the EFTA area) have set the validity at 365 days from the second dose, whereas Croatia [36] and Italy [37] have opted for 270 day validity (9 months), although as of this writing, the Italian government is reportedly mulling an extension to 1 year. If the duration were set at 6 months, that could lead to wealthy countries offering third doses as boosters before developing countries can immunize their citizens with even a first round [38]. Furthermore, even in countries that can rely on a quantity of vaccine doses sufficient to meet the needs of the entire population, vaccination certification requirements will have to take into account those who cannot be vaccinated through no fault of their own, due to medical conditions [39].

In our opinion, the alternation of the three certifications satisfies the principle of non-discrimination. In fact, those who possess the pass can travel without further obligations, and those who do not have it can travel, but under the conditions set by the states of arrival, such as quarantine. The restrictions codified in the COVID Digital Certificate appear reasonably balanced because they protect both the freedom of movement of European citizens and the right of everyone to decide for their own body, as well as health.

As it is well-known, the vaccine made in December 2020 by Pfizer and BioNTech (international nonproprietary name: tozinameran), sold under the brand name Comirnaty, is the first fully tested means of immunization approved for emergency use [40], and targeted studies focused on safety, immunogenicity and the rate of protection produced reassuring results [41]. Studies have shown that antibodies after infection last for several months [42], while the duration of immunity acquired with the vaccine is still uncertain [43]. In fact, even the vaccinated person can get sick, usually not seriously, and can be a source of contagion, albeit to a lesser degree. Therefore, as mentioned earlier, vaccination does not necessarily rule out that the individual can host and pass on the virus [44]. Furthermore, the degree of vaccination effectiveness against certain variants, both the ones already spreading and those that may arise in the future [45], is unclear. At the time being, however, there is little doubt that the anti-COVID-19 vaccine constitutes the most important tool for the protection of individual and collective health. Moreover, the free movement of vaccinated people is reconciled with the objective of protecting public health only if the vaccinated account for a significant percentage of the population overall [46]. Not even a serological test that reflects the amount of antibodies present in the blood can rule out the risk of a new infection and disease, even though it can strongly reduce it. Moreover, in this case, the duration of the acquired natural immunity is not certain. The serological test, on the other hand, only expresses humoral immunity, but does not measure cellular immunity (B cells, T lymphocytes and macrophages) as well as immunological memory [47]. The polymerase chain reaction (PCR) test, on the other hand, only certifies the absence of infection at the time it is carried out, but it does not rule out false negatives nor, above all, the possibility that the person may become ill immediately after having undergone testing with a negative result [48]. The tests currently available (rapid antigenic tests and PCR tests) have different degrees of sensitivity and are moderately invasive. The issue of invasiveness could pose a problem for children (currently excluded from vaccination) if they need to be tested repeatedly in the short term. Testing is certainly valuable for tracing purposes, particularly when it is necessary to check the health conditions of individuals with respect to a specific event, such as gatherings, parties, ceremonies and the like. In reality, only the certificate of recovery (which cannot be issued before the eleventh day from the first positive test result and is valid for 180 days) can ensure that the patient has both acquired immunity and is not a vehicle for infection. Further critical issues of ethical nature could arise from organizational and allocation-related problems, such as the difficulty of ensuring the availability of enough vaccines, which was highlighted by EMA [49], in a timely fashion. Besides, managing the enormous demand for COVID tests is in itself problematic, given the need to guarantee widespread usability at low costs or free of charge and to ensure continuity of supply. Furthermore, the psychological element should not be overlooked. Since it is not possible to completely eliminate the risk of contagion, the mere possession of the COVID Digital Certificate could generate a false sense of security, leading to reckless behaviors that could endanger the health of the individual and of those with whom he or she comes into contact [50]. Another risk worth considering is the abrupt failure of computer systems, either as a result of a virus or cybercrimes. In such an eventuality, government authorities in charge of managing the information could not carry out the checks and, therefore, free movement would be suspended, causing damage to citizens, and it might be necessary to get back to a travel-and-quarantine approach, with all the impractical aspects it entails, compared to one based on digital certification, verifiable within seconds. For example, a person who for any reason is abroad may not be in a position to return to his or her country if, due to a possible temporary disabling of computer systems linked to the control framework, it became impossible to issue or check the validity of the certificate, or they would have to quarantine to be cleared for traveling. Finally, it is worth bearing in mind that all forms of COVID pass, although not solely tied to vaccination such as that developed at European level, are bound to incentivize vaccination uptake. One may, therefore, wonder whether that is ethically acceptable or instead may be an unacceptable form of coercion, detrimental to the right to free self-determination, which is guaranteed for any medical treatment, thus coming to resemble a sort of roundabout coercion. In our view, however, such an incentivizing purpose appears to be completely legitimate both from an ethical and practical point of view, and is fundamentally driven by the fact that the free movement of vaccinated individuals is feasible with a limited and reasonable risk only if, as mentioned earlier, the vaccinated account for a relevant percentage of the population. It should therefore be explicitly declared, in order to defuse the accusation of pursuing untold objectives, i.e., compulsory vaccination.

## 7. Conclusions

By establishing a common system of interoperable certifications that can be used in all Member States, the European green pass legislation introduces a common system that prevents national authorities from putting in place restrictions on the right of movement in an inconsistent and uneven fashion. The COVID Digital Certificate allows for the possibility of traveling, albeit conditioned, which constitutes an indisputable advantage compared to an absolute and indiscriminate prohibition, which would severely penalize citizens and would likely be deemed intolerably intrusive. Naturally, the COVID Digital Certificate is justified by the exceptional nature of the current pandemic scenario, after which it should be immediately discontinued. Ultimately, this form of COVID pass appears to be fully legitimate to foster free movement, without disproportionately constraining such a right for the sake of health protection. Furthermore, such a framework requires widespread, thorough and understandable information campaigns, planned by experts that clearly and truthfully highlight the reasons, conditions, opportunities, purposes and limits of this documentation, in order to raise awareness among the population as to the distinctive traits of the various certifications contained in the COVID pass and the relevant differences in terms of safety, reliability and effectiveness. Only if these conditions are met will it be possible to stave off the risk that the COVID Digital Certificate may result in a false sense of security, leading to the ill-advised abandonment or loosening of non-pharmacological measures for stemming the pandemic, such as social distancing, hand hygiene and mask wearing.

## Figures and Tables

**Table 1:** Main characteristics of COVID passes.

(1) Digital and/or paper based (2) QR code (3) Free of charge (4) In the national language of issuing country and English (5) Safe and forgery-proof (6) Acknowledged as valid in all EU member states

## References

[1] European Centre for Disease Prevention and Control Daily Data. Issued on 18 August 2021. https://www.ecdc.europa.eu/en/cases-2019-ncov-eueea.

[2] Marinelli E., Busardò F.P., Zaami S. (2020). Intensive and Pharmacological Care in Times of COVID-19: A “Special Ethics” for Emergency?. BMC Med. Ethics.

[3] Montanari Vergallo G., Rinaldi R., Piersanti V., Tini A., Del Rio A. (2021). Is the Right to Abortion at Risk in Times of COVID-19? The Italian State of Affairs within the European Context. Medicina.

[4] European Parliament Resolution of 24 November 2020 on the Schengen System and Measures Taken during the COVID-19 Crisis (2020/2801(RSP)). https://www.europarl.europa.eu/doceo/document/TA-9-2020-0315_EN.pdf.

[5] European Commission COVID-19 Guidance on the Implementation of the Temporary Restriction on Non-Essential Travel to the EU, on the Facilitation of Transit Arrangements for the Repatriation of EU Citizens, and on the Effects on Visa Policy. Issued on 30 March 2020. https://ec.europa.eu/home-affairs/sites/default/files/what-we-do/policies/european-agenda-migration/20200330_c-2020-2050-report_en.pdf.

[6] Council Recommendation (EU) 2020/912 of 30 June 2020 on the Temporary Restriction on Non-Essential Travel into the EU and the Possible Lifting of Such Restriction. https://eur-lex.europa.eu/legal-content/EN/TXT/PDF/?uri=CELEX:32020H0912&from=EN.

[7] Schlagenhauf P., Patel D., Rodriguez-Morales A.J., Gautret P., Grobusch M.P., Leder K. (2021). Variants, Vaccines and Vaccination Passports: Challenges and Chances for Travel Medicine in 2021. Travel Med. Infect. Dis..

[8] Fontanet A., Autran B., Lina B., Kieny M.P., Karim S.S.A., Sridhar D. (2021). SARS-CoV-2 Variants and Ending the COVID-19 Pandemic. Lancet.

[9] Wilf-Miron R., Myers V., Saban M. (2021). Incentivizing Vaccination Uptake: The “Green Pass” Proposal in Israel. JAMA.

[10] List of EU Countries Issuing/Asking for Vaccination Certificates. Issued on 4 February 2021. https://www.schengenvisainfo.com/news/list-of-eu-countries-issuing-asking-for-vaccination-certificates/.

[11] Palmer S. EU Outlines Proposed COVID ‘Vaccine Passport’ That Could Boost Travel This Summer. Issued on 17 March 2021. https://www.euronews.com/travel/2021/03/17/eu-plans-to-boost-summer-travel-with-its-proposal-for-a-digitalvaccine-passport.

[12] Law Decree n.52, (12). Recante Misure Urgenti per la Qraduale Ripresa Delle Attività Economiche e Sociali Nel Rispetto Delle Esi-Genze di Contenimento Della Diffusione Dell’epidemia da COVID-19. Issued on 22 April 2021. https://temi.camera.it/leg18/temi/misure-urgenti-per-la-graduale-ripresa-delle-attivit-economiche-e-sociali-nel-rispetto-delle-esigenze-di-contenimento-della-diffusione-dell-epidemia-da-covid-19.html.

[13] Greely H.T. (2020). COVID-19 Immunity Certificates: Science, Ethics, Policy, and Law. J. Law Biosci..

[14] (2021). Europol Warning on the Lllicit Sale of False Negative COVID-19 Test Certificates. https://www.europol.europa.eu/newsroom/news/europol-warning-illicit-sale-of-false-negative-covid-19-test-certificates.

[15] Regulation (EU) 2021/954 of the European Parliament and of the Council of 14 June 2021 on a Framework for the Issuance, Verification and Acceptance of Interoperable COVID-19 Vaccination, Test and Recovery Certificates (EU Digital COVID Certificate) with Regard to Third-Country Nationals Legally Staying or Residing in the Territories of Member States during the COVID-19 Pandemic. http://www.europeanmigrationlaw.eu/en/articles/news/regulation-eu-2021954-eu-digital-covid-certificate-third-country-nationals-legally-staying-or-residing-in-the-territories-of-member-states.html.

[16] Sharun K., Tiwari R., Dhama K., Rabaan A.A., Alhumaid S. (2021). COVID-19 Vaccination Passport: Prospects, Scientific Feasibility, and Ethical Concerns. Hum. Vaccin. Immunother..

[17] Cardenas N.C. (2021). Advancing Strategic Policy on European Union Digital COVID-19 Certificate. J. Public Health.

[18] Council of Europe (2020). Information Documents SG/Inf(2020)11, Respecting Democracy, Rule of Law and Human Rights in the Framework of the COVID-19 Sanitary Crisis. A Toolkit for Member States. https://rm.coe.int/sg-inf-2020-11-respecting-democracy-rule-of-law-and-human-right.

[19] (2020). COE Committee on Bioethics (DH-BIO) Statement on Human Rights Considerations Relevant to the COVID-19 Pandemic. https://rm.coe.int/dh-bio-2021-7-final-statement-vaccines-e/1680a259dd.

[20] (2020). EU Commission, Recommendation on a Common Union Toolbox for the Use of Technology and Data to Combat and Exit from the COVID-19 Crisis, in Particular Concerning Mobile Applications and the Use of Anonymised Mobility Data. https://op.europa.eu/en/publication-detail/-/publication/1e8b1520-7e0c-11ea-aea8-01aa75ed71a1/language-en.

[21] EU Commission (2020). Guidance on Apps Supporting the Fight against COVID-19 Pandemic in Relation to Data Protection. https://ec.europa.eu/info/sites/default/files/5_en_act_part1_v3.pdf.

[22] Mbunge E., Dzinamarira T., Fashoto S.G., Batani J. (2021). Emerging Technologies and COVID-19 Digital Vaccination Certificates and Passports. Public Health Pract..

[23] Montanari Vergallo G., Zaami S., Marinelli E. (2021). The COVID-19 Pandemic and Contact Tracing Technologies, between Upholding the Right to Health and Personal Data Protection. Eur. Rev. Med. Pharmacol. Sci..

[24] Darbyshire T. (2020). Do We Need a Coronavirus (Safeguards) Act 2020? Proposed Legal Safeguards for Digital Contact Tracing and Other Apps in the COVID-19 Crisis. Patterns.

[25] Parliamentary Assembly Resolution 2361 (2021). COVID-19 Vaccines: Ethical, Legal and Practical Considerations. Issued on 27 January 2021. https://pace.coe.int/en/files/29004/html.

[26] Council of Europe Committee on Social Affairs, Health and Sustainable Development. COVID-19 Vaccines: Ethical, legal and Practical Considerations, Doc. 15212. Issued on 11 January 2021. https://pace.coe.int/pdf/6e68aa5b4c00953f2bc05e97e50949719ff2051bc05b1cf3a6241e2e19a3ab0c/doc.%2015212.pdf.

[27] Comitato Nazionale per la Bioetica Italian National Bioethics Committee. I Vaccini e COVID-19: Aspetti Etici per la Ricerca, il Costo e la Distribuzione. Issued on 27 November 2020. https://bioetica.governo.it/media/4115/p140_2020_vaccini-e-covid19_it.pdf.

[28] Aranzales I., Chan H.F., Eichenberger R., Hegselmann R., Stadelmann D., Torgler B. (2021). Scientists Have Favorable Opinions on Immunity Certificates but Raise Concerns Regarding Fairness and Inequality. Sci. Rep..

[29] Gensabella Furnari M. Vaccini e COVID-19: Aspetti Etici per la Ricerca, il Costo e la Distribuzione. Giustizia Insieme. Issued on 7 January 2021. https://www.giustiziainsieme.it/it/diritto-dell-emergenza-covid-19/1487-note-a-margine-del-parere-del-comitato-nazionale-per-la-bioetica-i-vaccini-e-covid-19-aspetti-etici-per-la-ricerca-il-costo-e-la-distribuzione-di.

[30] Subbaraman N. (2021). How Do Vaccinated People Spread Delta? What the Science Says. Nature.

[31] Brown R.C.H., Kelly D., Wilkinson D., Savulescu J. (2021). The Scientific and Ethical Feasibility of Immunity Passports. Lancet Infect. Dis..

[32] Baylis F., Kofler N. (2021). A Public Health Ethic Should Inform Policies on COVID-19 Immunity Passports. Lancet Infect. Dis..

[33] European Commission Questions and Answers—EU Digital COVID Certificate. Issued on 1 June 2021. https://ec.europa.eu/commission/presscorner/detail/en/QANDA_21_2781.

[34] Coronavirus Brussels. Brussels Information Portal on the Coronavirus. https://coronavirus.brussels/en/request-your-covid-certificate-to-travel-covid-safe/.

[35] Federal Office of Public Health FOPH Coronavirus: COVID Certificate. https://www.bag.admin.ch/bag/en/home/krankheiten/ausbrueche-epidemien-pandemien/aktu.

[36] EU Digitalna Poturda (EU) Vaccination Certificate. Frequently Asked Questions. https://www.eudigitalnacovidpotvrda.hr/en/faq.

[37] Certificazione verde COVID-19. EU Digital COVID Certificate. https://www.dgc.gov.it/web/faq.html#infgen.

[38] De Miguel Beriain I., Rueda J. (2020). Immunity Passports, Fundamental Rights and Public Health Hazards: A Reply to Brown et al. J. Med. Ethics.

[39] Voo T.C., Clapham H., Tam C.C. (2020). Ethical Implementation of Immunity Passports During the COVID-19 Pandemic. J. Infect. Dis..

[40] Ball P. (2021). The Lightning-Fast Quest for COVID Vaccines–And What It Means for Other Diseases. Nature.

[41] Dai L., Gao G.F. (2021). Viral Targets for Vaccines against COVID-19. Nat. Rev. Immunol..

[42] Wajnberg A., Amanat F., Firpo A., Altman D.R., Bailey M.J., Mansour M., McMahon M., Meade P., Mendu D.R., Muellers K. (2020). Robust Neutralizing Antibodies to SARS-CoV-2 Infection Persist for Months. Science.

[43] Dan J.M., Mateus J., Kato Y., Hastie K.M., Yu E.D., Faliti C.E., Grifoni A., Ramirez S.I., Haupt S., Frazier A. (2021). Immunological Memory to SARS-CoV-2 Assessed for up to 8 Months after Infection. Science.

[44] Italian Institute of Health Rapporto ISS COVID-19, Indicazioni ad Interim Sulle Misure di Prevenzione e Controllo Delle Infe-zioni da SARS-CoV-2 in Tema di Varianti e Vaccinazione Anti-COVID-19. Issued on 13 March 2021. https://www.iss.it/documenti-in-rilievo/-/asset_publisher/btw1J82wtYzH/content/rapporto-iss-covid-19-n.-4-2021-.

[45] Brazal A.M. (2021). Inoculation Now or Later? Lower Efficacy and Vaccine Passport Concerns. J. Public Health.

[46] Brown R.C.H., Savulescu J., Williams B., Wilkinson D. (2020). Passport to Freedom? Immunity Passports for COVID-19. J. Med. Ethics.

[47] Nehme M., Stringhini S., Guessous I. (2020). SEROCoV-Pop Study Team, null Perceptions of Immunity and Vaccination Certificates among the General Population: A Nested Study within a Serosurvey of Anti-SARS-CoV-2 Antibodies (SEROCoV-POP). Swiss Med. Wkly..

[48] Baylis F., Kofler N. COVID-19 Immunity Testing: A Passport to Inequity, Issues in Science and Technology. Issued on 29 April 2020. https://issues.org/covid-19-immunity-testing-passports/#:~:text=COVID-19%20Immunity%20Testing%3A%20A%20Passport%20to%20Inequity%20By,best%20to%20ease%20restrictions%20and%20reopen%20the%20economy.

[49] Phelan A.L. (2020). COVID-19 Immunity Passports and Vaccination Certificates: Scientific, Equitable, and Legal Challenges. Lancet.

[50] Drury J., Mao G., John A., Kamal A., Rubin G.J., Stott C., Vandrevala T., Marteau T.M. (2021). Behavioural Responses to COVID-19 Health Certification: A Rapid Review. BMC Public Health.

